# Long-term retention and predictors of attrition for key populations receiving antiretroviral treatment through community-based ART in Benue State Nigeria: A retrospective cohort study

**DOI:** 10.1371/journal.pone.0260557

**Published:** 2021-11-30

**Authors:** Olujuwon Ibiloye, Plang Jwanle, Caroline Masquillier, Sara Van Belle, Ekere Jaachi, Olubunmi Amoo, Ahmed Isah, Temiwoluwa Omole, Jay Osi Samuel, Josefien van Olmen, Lutgarde Lynen, Prosper Okonkwo, Tom Decroo

**Affiliations:** 1 Institute of Tropical Medicine, Antwerp, Belgium; 2 APIN Public Health Initiatives, Abuja, Nigeria; 3 University of Antwerp, Antwerp, Belgium; 4 Research Foundation Flanders, Brussels, Belgium; University of Pretoria, SOUTH AFRICA

## Abstract

**Background:**

Key populations (KP) are disproportionately infected with HIV and experience barriers to HIV care. KP include men who have sex with men (MSM), female sex workers (FSW), persons who inject drugs (PWID) and transgender people (TG). We implemented three different approaches to the delivery of community-based antiretroviral therapy for KP (KP-CBART) in Benue State Nigeria, including One Stop Shop clinics (OSS), community drop-in-centres (DIC), and outreach venues. OSS are community-based health facilities serving KP only. DIC are small facilities led by lay healthcare providers and supported by an outreach team. Outreach venues are places in the community served by the outreach team. We studied long-term attrition of KP and virological non-suppression.

**Method:**

This is a retrospective cohort study of KP living with HIV (KPLHIV) starting ART between 2016 and 2019 in 3 0SS, 2 DIC and 8 outreach venues. Attrition included lost to follow-up (LTFU) and death. A viral load >1000 copies/mL showed viral non-suppression. Survival analysis was used to assess retention on ART. Cox regression and Firth logistic regression were used to assess risk factors for attrition and virological non-suppression respectively.

**Result:**

Of 3495 KPLHIV initiated on ART in KP-CBART, 51.8% (n = 1812) were enrolled in OSS, 28.1% (n = 982) in DIC, and 20.1% (n = 701) through outreach venues. The majority of participants were FSW—54.2% (n = 1896), while 29.8% (n = 1040), 15.8% (n = 551) and 0.2% (n = 8) were MSM, PWID, and TG respectively. The overall retention in the programme was 63.5%, 55.4%, 51.2%, and 46.7% at 1 year, 2 years, 3 years, and 4 years on ART. Of 1650 with attrition, 2.5% (n = 41) died and others were LTFU. Once adjusted for other factors (age, sex, place of residence, year of ART enrollment, WHO clinical stage, type of KP group, and KP-CBART approach), KP-CBART approach did not predict attrition. MSM were at a higher risk of attrition (vs FSW; adjusted hazard ratio (aHR) 1.27; 95%CI: 1.14–1.42). Of 3495 patients, 48.4% (n = 1691) had a viral load test. Of those, 97.8% (n = 1654) were virally suppressed.

**Conclusion:**

Although long-term retention in care is low, the virological suppression was optimal for KP on ART and retained in community-based ART care. However, viral load testing coverage was sub-optimal. Future research should explore the perspectives of clients on reasons for LTFU and how to adapt approach to CBART to meet individual client needs.

## Introduction

The World Health Organization (WHO) guidelines on HIV prevention, diagnosis, care, and treatment for key populations (KP) identified female sex workers (FSW), men who have sex with men (MSM), prisoners, persons who inject drugs (PWID), and transgender people (TG) as key populations, at substantial risk for becoming infected with HIV and also potentially driving HIV transmission [1]. The burden of HIV among KP is disproportionately high compared to that of the general population. In 2020, KP and their sex partners accounted for 65% of new HIV infections globally and 39% in sub-Saharan Africa [2]. Compared to the general population, The risk of HIV acquisition is 25, 35, 26 and 34 times times higher for MSM, PWID, FSW and TG respectively [2].

The HIV epidemic in Nigeria is mixed, meaning that HIV prevalence is high in both the KP and general population. Findings from the various surveys showed that KP have a greater HIV burden compared to the general population [3–6]. In 2009, PWID, MSM, and FSW constitute about 3.4% of the adult population and yet almost 23% of all new HIV infections in the country occur in this subgroup [3]. The 2020 HIV integrated biological and behavioural surveillance survey (IBBSS) estimated the HIV prevalence for FSW, MSM, PWID, and TG to 16.7%, 20.9%, 6.2%, and 9.5%, respectively [6], much higher than the 2018 national HIV prevalence of 1.4% among those between 15–49 years old [5]. Despite the high HIV burden among KP in Nigeria, antiretroviral treatment (ART) coverage as of 2020 is still low among FSW (23.7%), PWID (23%), TG (19.5%), and MSM (26.3%) [6].

One of the barriers to effective treatment for KP living with HIV (KPLHIV) is poor access to care. Moreover, retention on ART is a major challenge for many ART programmes, across different settings and populations [7]. Several studies have identified individual, social and environmental barriers to adherence and retention in ART programmes [8–11]. Predictors of disengagement from ART care include male sex, lower educational status, unemployment, advanced HIV disease, distance from a clinic, and non-disclosure of HIV status [7]. More than in the general population, stigma and discrimination, lack of community empowerment, gender inequality, violence, and criminalizing laws and policies hamper access to HIV care for KP [1]. A study conducted in Uganda among FSW identified the following barriers to HIV care: perceived stigma, fear to be seen at outreach HIV clinics, fear and myths about antiretroviral therapy, lack of time to attend clinic, and financial constraints [12].

To overcome barriers to access and engagement in care, the WHO recommended community-based approaches to ART care [1, 7]. Moreover, the WHO proposed National HIV programmes to engage KP to better adapt ART delivery to their needs and as such improve HIV treatment outcomes [1, 7]. These recommendations align well with the Nigerian Government’s National HIV and AIDS Strategic Framework 2017–2022 [3] that outlined community-based ART service delivery models, multi-month drug refill, and peer-led and health care worker (HCW)-led approaches for ART delivery to improve access to care and treatment outcomes among KPLHIV.

A trial and a prospective study in Tanzania showed high retention rates in ART care among FSW receiving ART through a community-based ART programme for Key populations (KP-CBART) [13, 14] However, there are only few studies on how this evidence is translated to less-resourced routine care [9, 15]. These studies reported retention rate in KP-CBART models between 6–12 months on ART. One study documented retention rate of about 73.0% for MSM, FSW, PWID, and TG in a CBART model [16] and another reported 72.2% retention rate at 6 months on ART for MSM receiving ART through a community-based health centre in Nigeria [17]. Furthermore, there is no evidence on long-term treatment outcomes among KP attending KP-CBART.

In Benue State, KP-CBART includes provision of ART through three different approaches. Hereafter referred to as KP-CBART approach. There are three community-based health centres, also known as “One Stop Shop clinic (OSS)”, two drop-in-centres (DIC), and eight community outreach venues. The OSS is a community-based health centre that provides comprehensive HIV care to KP. The Drop-in-Centre is a mini-OSS led by lay healthcare providers. As the term shows, outreach venues do not rely on a physical health infrastructure but deliver services in agreed locations within the community. In community venues medical health care workers (HCW) liaise with a network of KP-led and KP-competent civil society organizations. Both DIC and outreach venues are served by a mobile multidisciplinary health team that ensures medical care.

The aim of this study is to evaluate the performance of KP-CBART in Benue State, in Nigeria using routine programme data. We described the characteristics of KP stratified by KP-CBART approach, explored the HIV virological suppression and attrition rates and the predictors of these outcomes.

## Methods

### Study design

This is a retrospective cohort study of KP-CBART using routine programmatic data and the report is based on the reporting guidelines of studies conducted using observational routinely-collected health Data (RECORD) [18].

### The Benue State community-based ART service delivery model for key populations

Benue State has a KP-CBART programme that is part of the national HIV programme since 2016. This model of care was designed for HIV-positive MSM, FSW, PWID, and TG to increase their access to HIV prevention, care, and treatment in the state and to improve their treatment outcomes. This programme is implemented by the Agency for the Control of AIDS, Ministry of Health, and implementing partners with support from PEPFAR through the Centre for Disease Control.

The three OSS are community-based health centres that provide comprehensive HIV services strictly to KP in an environment that aims at being free of stigma and discrimination. The OSS is located in the urban setting. relies on a team of HCW, including medical staff providing HIV testing and clinical care, and lay staff or peer educators for adherence counselling, client tracing, support group meetings, and escort services to the Tuberculosis (TB) treatment centres. HCW participated in a comprehensive ART training including adherence to build their capacity to provide HIV care and treatment services to patients. Besides clinical activities, the OSS serves as a meeting place for KP to engage in recreational activities. OSS also have outreach teams, which consists of an ART clinician, a peer educator, a triage nurse, and/or pharmacist. These outreach teams ensure a comprehensive package of outreach activities at DIC and community outreach venues.

The two DIC are mini-OSS and are safe places where KP can meet to socialize, make friends and also access clinical activities when the outreach team is around. To ensure easy access, DIC are located close to “hotspots” (hotels, brothels, club houses, among others) and are located in semi-urban areas. The main difference between OSS and DIC is that OSS are led by medical HCW while DIC are led by both a Nurse Case Manager and lay healthcare providers (community facilitators). Community facilitators are actively involved in the planning and conduct of outreach, support group meetings, and drug refill by proxy. The DIC do not have their own ART clinician but rely on the outreach team for clinical care. Similar to the OSS, DIC offer a comprehensive package of services, such as HIV-testing services, adherence counselling, condom distribution, ART initiation, ART refill and referral if needed.

Community outreach venues are locations within the community where the outreach teams and community facilitators from the OSS provide ART care to KPLHIV. These venues are located in communities without operational DIC or OSS. OSS and DIC are located in urban and semi-urban areas while outreach venues are mostly found in rural communities. The community facilitators mobilise their peers for HIV diagnosis, ART initiation, ART refill, and other services while treatment officers plan HIV outreach services. In addition, within communities outreach teams visit hotspots to sensitize and mobilise clients for HIV testing and ART initiation. Community facilitators provide voluntary HIV testing and counselling. Community facilitators are engaged by civil society organizations and they work within the community, pay home visits, and render services at outreach venues. Some community facilitators and treatment officers are KP (eg peer educators) and rely on their own experiences of living with HIV. Community facilitators were trained by KP-led and KP-friendly civil society organizations to provide HIV services. They receive HIV counselling and testing (HTC) training for a minimum of 10 days.

Stable patients on ART, adherent to medication/clinic appointments, can benefit from drug pick-up by proxy after 6–12 months of clinical stability. Drug pick-up by proxy is available in OSS, DIC and outreach venues. It involves ART drug dispensing through a diverse group of lay providers (community facilitators and treatment partners) to patients on ART who in turn provide ART to the client.

Patients that are lost to follow-up (LTFU) are tracked through phone calls and home visits. LTFU refers to no clinical contact or drug refill from any of the KP-CBART approaches for more than 28 days since the last expected contact. Lists of patients LTFU are generated using appointment registers at the end of each scheduled outreach/clinic for immediate tracking by the community facilitators.

Patients’ viral load status is assessed in line with the 2020 National Guidelines for HIV Prevention, Care, and Treatment in Nigeria. Viral load testing is conducted for HIV infected persons at 6 and 12 months on antiretroviral treatment, and thereafter annually. In the KP-CBART, clients’ clinic and ART refill appointment are structured to align with viral load testing schedule to ensure that clients do not miss their viral load test.

### Study population

Adult (18 years or older) KPLHIV who initiated ART in the KP-CBART programme between January 1, 2016 and December 31, 2019 and who were followed up until January 31, 2021 in Benue State Nigeria. Clients were initiated on ART based on the Test and Treat criteria (ART initiation regardless of CD4 count or WHO clinical staging), coherent with the Nigeria Guidelines for HIV Prevention and Treatment.

### Data collection and analysis

Individual-level data of patients enrolled into the KP-CBART programme between January 2016 and December 2019 were extracted from the electronic medical record (EMR) into a standardized excel-based data extraction template. EMR data were triangulated with patient record in the folders and registers for missing data. Variables that were extracted included demographic data (age, sex, education, occupation, residence), clinical variables (HIV status, date of HIV diagnosis, linkage to care, ART status, WHO stage, TB status, viral load suppression) and treatment outcomes (LTFU, dead, transferred out, active in care).

Attrition referred to those who died (either by direct observation or by family or close contact report) or were LTFU. LTFU was defined as no clinical contact or drug refill from any of the above described approaches for more than 28 days since the last expected contact. Retention in care on ART was defined as the proportion of patients that are active in care and on ART, among those who commenced ART and those transferred out to another HIV programme or facility. Virological non-suppression was defined as having a last viral load higher than 1000 copies/mL.

Baseline characteristics were summarised stratified by KP-CBART approach. Proportions were calculated for categorical variables while medians and interquartile ranges were calculated for continuous variables. The Chi-squared test was used to identify associations between categorical variables.

We employed bivariable and multivariable Cox regression to estimate the association between explanatory variables and attrition on ART. Patients transferred out were censored on the date they were transferred out. Patients retained in care at the end of the study period were censored on the last contact with the KP-CBART programme. We considered patients who were LTFU and those that died as having experienced the event and were censored on the date of their last ART refill appointment or on the date of their death. In patients with a viral load result, Firth’s logistic regression for rare events was used to determine the association between explanatory variables and virological non-suppression. Also, we estimated factors associated with not having a viral load test done among patients retained in care at the end of the study period. For both the Cox and logistic regression first saturated multivariable models were constructed. These were then stepwise simplified until only the variables of interest (KP-CBART approach and KP type) and variables associated with the event remained. Kaplan–Meier techniques [19] were employed to estimate retention by KP-CBART approach. The Log-rank test was used to estimate the differences in Kaplan-Meier curves between subgroups. Statistical significance was based on p-value < 0.05. Statistical analysis was done using SPSS (version 16) and Stata (version 16.1) software.

### Ethics

The study was approved by the Institutional Research Board of APIN Public Health Initiatives (IRB022-FR), Benue State Ministry of Health and Human Services, and the Institute of Tropical Medicine Antwerp (1503/21), providing a waiver for the obligation to seek informed consent. Study participants were protected as all data were anonymized before accessing them. The database did not include personal identifiers such as names or addresses.

## Results

### Baseline characteristics

In this study, we extracted data for 3495 KPLHIV initiated on ART in the CBART programme between 2016 and 2019. An estimated 51.8% (n = 1812) of participants were enrolled in OSS while 28.1% (n = 982) were enrolled in the community DIC and 20.1% (n = 701) through community outreach (Table 1).

**Table 1:** Baseline characteristics by approach among key populations enrolled on ART between 2016–2019 and attending community-based ART services in Benue State, Nigeria.

	Total		OSS		DIC		Outreach		
	N	(%)	N	(%)	N	(%)	N	(%)	p-value $
**Total**	3495		1812	100	982		701		
**Sex**									<0.001
Female	2118	60.6	1146	63.2	538	54.8	434	61.9	
Male	1377	39.4	666	36.8	444	45.2	267	38.1	
**Age at enrolment**									<0.001
<25	252	7.2	146	8.1	60	6.1	46	6.6	
25-<40	2328	66.6	1256	69.3	635	64.7	437	62.3	
40-<55	844	24.1	370	20.4	268	27.3	206	29.4	
≥ 55	71	2	40	2.2	19	1.9	12	1.7	
**Place of residence**									<0.001
Rural	1321	37.8	213	11.8	903	92	205	29.2	
Semi-urban	475	13.6	21	1.2	0	0	454	64.8	
Urban	1595	45.6	1550	85.5	10	1	35	5	
No data	104	3	28	1.5	69	7	7	1	
**Year of ART enrolment**									<0.001
2016	215	6.2	96	5.3	47	4.8	72	10.3	
2017	1510	43.2	740	40.8	550	56	220	31.4	
2018	881	25.2	500	27.6	168	17.1	213	30.4	
2019	889	25.4	476	26.3	217	22.1	196	28	
**WHO stage at ART start**									0.005
1	3179	91	1612	89	947	96.4	620	88.4	
2	124	3.5	80	4.4	18	1.8	26	3.7	
3 or 4	23	0.7	13	0.7	5	0.5	5	0.7	
No data	169	4.8	107	5.9	12	1.2	50	7.1	
**Key population**									<0.001
FSW	1896	54.2	1041	57.5	458	46.6	397	56.6	
MSM	1040	29.8	475	26.2	350	35.6	215	30.7	
PWID	551	15.8	289	15.9	174	17.7	88	12.6	
TG	8	0.2	7	0.4	0	0	1	0.1	

*$ Chi-squared test, without category "No data".

OSS–One Stop Shop clinic, DIC–community drop-in-centre, FSW- female sex worker, MSM–men who have sex with men, PWID–person who inject drugs, TG–transgender people.

At baseline, the median age of participants was 34 years (IQR: 29–40) and more than half (60.6%, n = 2118) of all participants were female. About 45.6% (n = 1595) of the participants were living in the urban area while 13.6% (n = 475) and 37.8% (n = 1321) lived in semi-urban and rural areas respectively. The vast majority of participants were in the early stage of HIV disease at the time of diagnosis and enrolment: 91% (n = 3179) of them were diagnosed with WHO Stage 1 HIV disease.

The majority of participants enrolled in KP-CBART were FSW (54.2%, n = 1896), while 29.8% (n = 1040), 15.8% (n = 551) and 0.2% (n = 8) were MSM, PWID, and TG, respectively. In OSS and community outreach the proportion of KP that were FSW were nearly the same (57.5% vs 56.6%). The proportion of KP that were MSM (26.2%) in OSS was lower than in DIC (35.6%). Overall, only 0.2% (n = 8) TG were enrolled in the programme: 7 in DIC and 1 in community outreach. In community outreach the proportion (12.6%) of KP that were PWID was the lowest.

### Retention

Overall, retention in care was 63.5%, 55.4%, 51.2%, and 46.7% at 1 year, 2 years, 3 years, and 4 years on ART (Table 2). Of 1650 patients with attrition, 41 (2.5%) were reported as having died, while 97.5% (n = 1609) were LTFU.

**Table 2:** Retention by approach among key-populations enrolled on ART between 2016–2019 and attending community-based ART services in Benue State, Nigeria.

	OSS		DIC		Outreach		All models	
*Retention	%	(95%CI)	%	(95%CI)	%	(95%CI)	%	(95%CI)
12 months	59.30	57.0–61.6	62.5	59.4–65.5	75.8	72.4–78.9	63.5	61.9–65.1
24 months	51.40	49.0–53.8	56.7	53.5–59.8	64.2	60.4–67.8	55.4	53.8–57.2
36 months	46.3	43.8–48.8	55.7	52.5–58.8	57.6	53.5–61.6	51.2	49.4–52.9
48 months	38.9	35.8–42.2	55.0	51.6–58.1	53.6	49.1–57.9	46.7	44.7–48.8

OSS–One Stop Shop clinic, DIC–community drop-in-centre

*Kaplan Meir Survival Statistics were used to calculate the probability of retention at given time-points (12, 24, 36, and 48 on ART).

In the bivariable Cox regression analysis, age, sex, residence, year of ART initiation, KP type, and KP-CBART approach were significantly associated with attrition (Table 3, p-value< 0.05).

**Table 3:** Attrition and predictors of attrition among key-populations enrolled on ART between 2016–2019 and attending community-based ART services in Benue State, Nigeria.

	Total	Retained		Attrition		HR	[95%CI]	aHR	[95%CI]
	N	N	(%)	N	(%)				
**Total**	3495	1845	52.8	1650	47.2				
**Sex**								NS	
Female	2118	1144	54.0	974	46	Ref			
Male	1377	701	50.9	676	49.1	1.14**	[1.04,1.26]		
**Age at enrolment**									
<25	252	133	52.8	119	47.2	1.23*	[1.00,1.52]	1.2	[0.97,1.48]
25-<40	2328	1189	51.1	1139	48.9	1.28***	[1.13,1.44]	1.27***	[1.13,1.43]
40-<55	844	486	57.6	358	42.4	Ref		Ref	
≥ 55	71	37	52.1	34	47.9	1.14	[0.80,1.62]	1.01	[0.71,1.43]
**Place of residence**									
Semi-urban	475	310	65.3	165	34.7	Ref		Ref	
Rural	1321	731	55.3	590	44.7	1.52***	[1.28,1.81]	1.70***	[1.34,2.15]
Urban	1595	737	46.2	858	53.8	1.93***	[1.64,2.28]	1.98***	[1.52,2.60]
No data	104	67	64.4	37	35.6	1.08	[0.75,1.54]	1.16	[0.78,1.72]
**Year of ART enrolment**									
2016	215	124	57.7	91	42.3	Ref		Ref	
2017	1510	673	44.6	837	55.4	1.77***	[1.42,2.21]	1.68***	[1.35,2.10]
2018	881	450	51.1	431	48.9	1.66***	[1.32,2.10]	1.56***	[1.24,1.97]
2019	889	598	67.3	291	32.7	1.16	[0.91,1.48]	1	[0.79,1.28]
**WHO stage at ART enrolment**								NS	
1	3179	1719	54.1	1460	45.9	Ref			
2	124	77	62.1	47	37.9	0.73*	[0.55,0.98]		
3 or 4	23	13	56.5	10	43.5	0.74	[0.40,1.38]		
No data	169	36	21.3	133	78.7	3.02***	[2.52,3.61]		
**Key population**									
FSW	1896	1003	52.9	893	47.1	Ref		Ref	
MSM	1040	522	50.2	518	49.8	1.16**	[1.04,1.29]	1.27***	[1.14,1.42]
PWID	551	316	57.4	235	42.6	0.92	[0.80,1.07]	0.96	[0.83,1.11]
TG	8	4	50	4	50	1.38	[0.52,3.70]	1.59	[0.59,4.28]
**CBART approach**									
OSS	1812	865	47.7	947	52.3	Ref		Ref	
DIC	982	554	56.4	428	43.6	0.79***	[0.71,0.89]	0.88	[0.73,1.08]
Outreach	701	426	60.8	275	39.2	0.64***	[0.56,0.74]	1.03	[0.83,1.29]

* p < 0.05

** p < 0.01

*** p < 0.001.

NS: not significant.

OSS–One Stop Shop clinic, DIC–community drop-in-centre, FSW- female sex worker, MSM–men who have sex with men, PWID–person who inject drugs, TG–transgender people.

In the multivariable Cox regression analysis, there was no statistically significant difference between the risk of attrition and KP-CBART approach. In addition, sex and WHO staging were not significantly associated with attrition in multivariable analysis (p-value> 0.05). The hazard of attrition was higher among participants aged 25–39 years (vs those who were 40 years and older; adjusted hazard ratio (aHR) 1.27; 95%CI: 1.13–1.43), among MSM (vs FSW; aHR 1.27; 95%CI: 1.14–1.42), among participants started on ART in the 2017 (aHR 1.68; 95%CI: 1.35–2.10) and 2018 cohort (aHR 1.56; 95%CI: 1.24–1.97) as compared to those who started ART 2019, and among KP living in rural (aHR 1.70; 95%CI: 1.34–2.15) and urban areas (aHR 1.98; 95%CI: 1.52–2.60) as compared to those living in the semi-urban areas.

### Virological coverage and non-suppression

Of 3495 patients 48.4% (n = 1691) had a VL (Table 4). Of 1691 with a VL, 97.8% (n = 1654) had a viral load <1000 copies/mL.

**Table 4:** Virological outcomes among key-populations enrolled on ART between 2016–2019 and attending community-based ART services in Benue State, Nigeria.

	OSS		DIC		Outreach		Total	
**Viral load coverage (n = 3495)**	**N**	**%**	N	%	N	%	N	%
With VL result	833	45.9	535	55	323	46.1	1691	48.4
No VL test	979	54	447	46	378	53.9	1804	51.6
**Viral load suppression (n = 1691)**	** **	** **						
< 1000 copies/mL	814	97.7	529	99	311	96.3	1654	97.8
> 1000 copies/mL	19	2.3	6	1.1	12	3.7	37	2.2

OSS–One Stop Shop clinic, DIC–community drop-in-centre, VL- viral load.

S1 Table shows the factors associated with lack of viral load test in the KP-CBART approach. Predictors of lack of viral load test among patients that were retained in care at the end of the study period include being younger than 25 years old (aOR = 1.74, 95%CI: 1.02–2.96), 25–39 years old (aOR = 1.41, 95%CI: 1.02–1.96) and the type of CBART approach in which patients enrolled in (DIC- aOR = 0.25, 95%CI: 0.16–0.40), (Outreach–aOR = 2.59, 95%CI: 1.94–3.46).

In bivariable logistic regression, sex, place of residence, and WHO Stage at ART enrolment were significantly associated with virological non-suppression.

In the multivariable logistic regression model, virological non-suppression was associated with WHO Stage 2 (adjusted odds ratio (aOR) 3.76, 95%CI: 1.45–9.75) compared to WHO stage 1 at baseline. The type of KP population group and the KP-CBART approach were not associated with virological non-suppression (Table 5).

**Table 5:** Virological non-suppression and its predictors among key-populations enrolled on ART between 2016–2019 and attending community-based ART services in Benue State, Nigeria.

	Total	Virologically suppressed (<1000copies/mL)		Virologically non-suppressed (> = 1000cp/mL)		OR	[95%CI]	aOR [95%CI]	
	N	N	(%)	N	(%)				
**Total**	1691	1654	97.8	37	2.2				
**Sex**								NS	
Female	1047	1018	97.2	29	2.8	Ref			
Male	644	636	98.8	8	1.2	0.46*	[0.21,1.00]		
**Age at enrolment**								NS	
<25	116	111	95.7	5	4.3	2.9	[0.95,8.88]		
25-<40	1091	1067	97.8	24	2.2	1.35	[0.59,3.08]		
40-<55	447	440	98.4	7	1.6	Ref			
≥ 55-	37	36	97.3	1	2.7	2.41	[0.40,14.39]		
**Place of residence**								NS	
Semi-urban	230	222	96.5	8	3.5	Ref			
Rural	684	675	98.7	9	1.3	0.37*	[0.14,0.94]		
Urban	709	691	97.5	18	2.5	0.7	[0.31,1.60]		
No data	68	66	97.1	2	2.9	0.98	[0.23,4.14]		
**Year of ART enrolment**								NS	
2016	124	122	98.4	2	1.6	Ref			
2017	609	599	98.4	10	1.6	0.86	[0.21,3.46]		
2018	404	392	97	12	3	1.56	[0.40,6.16]		
2019	554	541	97.7	13	2.3	1.22	[0.31,4.78]		
**WHO stage at ART start**									
1	1606	1574	98	32	2	Ref		Ref	
2	68	63	92.6	5	7.4	4.20**	[1.64,10.72]	3.76**	[1.45,9.75]
3 or 4	14	14	100	0	0	NA		NA	
No data	3	3	100	0	0	NA		NA	
**Key population**									
FSW	919	892	97.1	27	2.9	Ref		Ref	
MSM	474	466	98.3	8	1.7	0.59	[0.27,1.29]	0.68	[0.31,1.49]
PWID	295	293	99.3	2	0.7	0.28	[0.08,1.02]	0.29	[0.08,1.09]
TG	3	3	100	0	0	NA		NA	
**CBART approach**									
OSS	833	814	97.7	19	2.3	Ref		Ref	
DIC	535	529	98.9	6	1.1	0.51	[0.21,1.25]	0.57	[0.23,1.41]
Outreach	323	311	96.3	12	3.7	1.68	[0.81,3.45]	1.60	[0.77,3.29]

* p < 0.05

** p < 0.01

*** p < 0.001.

NS—not significant, OSS–One Stop Shop clinic, DIC–community drop-in-centre, FSW- female sex worker, MSM–men who have sex with men, PWID–person who inject drugs, TG–transgender people, OR-odd ratio, aOR, adjusted odd ratio.

## Discussion

We studied long-term retention and predictors of attrition among KP receiving ART through KP-CBART in Benue State Nigeria. Attrition rates increased with increasing duration on ART, with less than half retained after 4 years since the start of treatment. Attrition mainly consisted of patients LTFU. Once adjusted for other predictors, type of KP or the approach to KP-CBART did not predict attrition. The high LTFU rate among KP enrolled into the CBART models explains the sub-optimal viral load coverage in the programme. However, among clients with recent viral test result, viral load suppression was optimal. The rate of viral suppression in the Benue KP-CBART model is higher compared to previous CBART studies among KP and the general population [16, 17, 20, 21]. Even though attrition is problematic, adherence among those retained in care and with VL test results in the KP-CBART is not compromised.

Our study showed data for all KP subgroups and showed data on long-term retention. Most previous CBART studies were designed for a specific KP subgroup, without data for retention after 12 months on ART [14, 17]. One study assessed a model serving all KP sub-groups but only reported early retention, with 73.2% retention after a median follow-up time of 7 months on ART [16]. Only one previous study assessed long-term retention on ART in KP-CBART: a retrospective cohort study from Ivory Coast showed a low level of retention among MSM and FSW on ART was 68% at 12 months, 55% at 24 months, and 47% at 36 months [9]. In Nigeria another study showed 56% LTFU after a median follow-up time of 12 months among MSM and TG receiving HIV care in community venues [8]. These study findings are coherent with our own findings, showing a low level of retention. One-year retention among KP in CBART was much lower than 98% retention in the general population served by CBART in Nigeria [21]. On the other hand retention in KP-CBART was comparable to retention in care in other vulnerable populations, such as children and adolescents in Nigeria [10]. One pediatric study reported an overall attrition of 49.4% among those under 17 years old and enrolled on ART [10].

The definition of LTFU in our study is more stringent compared to most studies that defined LTFU as being 2–6 months late for their last clinic appointment [8, 9, 16, 21], which may partially explain the difference in attrition reported between different studies. In our study retention in care worsened steadily with increasing duration on ART, with many LTFU before the end of the study period. Hence, the high proportion of LTFU cannot solely be explained by the LTFU definition. While some patients LTFU might have died, and while others probably truly stopped ART, some patients LTFU may in fact be in care elsewhere [22]. Indeed, previous studies showed that high mobility of KP, especially among FSW, explained poor retention in care [9, 23]. The high level of virological suppression among those retained suggests that clients do give importance to ART and adhere to treatment. The implementation of biometric systems, which allows correcting for silent transfers [24], at present falsely reported as LTFU, may be another step towards improving monitoring and reporting attrition in this highly mobile population of KP. However, how to assure confidentiality and privacy when collecting strictly personal data remains a challenge.

The three approaches to KP-CBART described in this study are examples of differentiated service delivery (DSD). DSD is defined as ‘‘a person-centred approach that simplifies and adapts HIV services across the cascade in ways that both serve the needs of people living with and vulnerable to HIV and optimize available resources in health systems” [7, 25]. In our study, we found that factors such as age, place of residence, year of ART enrolment, and type of KP were associated with attrition. These findings are consistent with previous studies on predictors of attrition among KPLHIV enrolled on ART [8, 16]. In Benue state, the choice of KP-CBART approach was dependent on clients’ place of residence because only one approach was available per geographical area. To improve adherence and retention on ART, there is need to modify the current models of differentiated service delivery to accommodate better (the individual clients’) needs and preferences. Qualitative research is needed to explore these needs and preferences. For example, how factors such as harassment of sex workers by security personnel and periodic relocation of HIV infected FSW after few months or years in a setting for better patronage does affect their engagement in HIV care needs to be explored. Possibly KP may benefit from further differentiation of ART service delivery (adaptation) in the program. Evidence has shown that provision of people-centred care (such as individualised adherence counselling, shared decision-making and planning for ART initiation, and supporting change in provider attitudes towards those who have interrupted their treatment) can improve HIV treatment outcomes [7, 26]. People-centred health services are ‘‘an approach to care that consciously adopts the perspectives of individuals, families and communities and sees them as participants and beneficiaries of trusted health systems that respond to their needs and preferences in humane and holistic ways” [7]. MSM are at higher risk of attrition compared to FSW. This can be explained by contextual factors that include greater stigma and discrimination that MSM face in the community [27]. Also, provision of ART to all the KP subgroups through the same model of ART care may have promoted stigma/discrimination between KP subgroups. Having a separate model of ART care for each of the KP subgroup may promote privacy and confidentiality and improve health outcomes. Therefore, we recommend that future qualitative research will also explore whether clients prefer ART delivery serving only one KP subgroup.

The strengths of this study include a large study cohort and long duration of follow-up in a non-research setting. The outcomes of this study reflect the real challenges influencing treatment outcomes of KP in the CBART. A major limitation of this study was that we could not verify the true outcomes of those reported as LTFU. Previous studies showed that some patients LTFU are underreported deaths, while a substantial proportion is active in care elsewhere, thus erroneously reported as LTFU [22]. We reported on a limited number of predictors, as data were not complete for occupation, marital status, level of education, and TB status at ART enrolment. There was selection bias due to sub-optimal viral load testing coverage in the programme and thereby limiting the validity of the viral load findings. A qualitative research is planned to explore the experiences and views of clients and to understand the facilitators and barriers to accessing VL testing in KP-CBART in Benue State. Findings from our study maybe transferable to other states in Nortcentral Nigeria. Similar studies across the country and in other countries will inform if our findings can be generalised to other settings.

## Conclusion

In conclusion, long-term attrition on ART is high among KP in CBART in Benue state, while virological suppression was high among those retained in care. How to reach best vulnerable groups remains unresolved. Further differentiation may be required, offering a true choice to individuals. Qualitative research should explore how to adapt KP-CBART for each of the KP subgroups. Moreover, the risks and benefits of innovative monitoring systems that correct for silent transfers should be studied.

## Supporting information

S1 File Inclusivity in global research. (DOCX)

S1 Table Lack of viral load test and its predictors among key-populations retained in care and attending community-based ART services in Benue State, Nigeria. (DOCX)
