# Supplementary material for: Long-term retention and predictors of attrition for key populations receiving antiretroviral treatment through community-based ART in Benue State Nigeria: A retrospective cohort study
Source: PLoS One. 2021 Nov 30;16(11):e0260557. doi: 10.1371/journal.pone.0260557 (PMC8631647; doi:10.1371/journal.pone.0260557)
Supplement: S1 Table — (DOCX) [file pone.0260557.s002.docx]

**S1 Table. Lack of viral load test and its predictors among key-populations retained in care and attending community-based ART services in Benue State, Nigeria**

|  | Total | With VL | | Without VL | | OR | [95%CI] | aOR | [95%CI] |
| --- | --- | --- | --- | --- | --- | --- | --- | --- | --- |
|  | N | N | (%) | N | (%) |  |  |  |  |
| **Total** | 1845 | 1578 | 85.5 | 267 | 14.5 |  |  |  |  |
| **Sex** |  |  |  |  |  |  |  | NS |  |
| Female | 1144 | 978 | 85.5 | 166 | 14.5 | Ref |  |  |  |
| Male | 701 | 600 | 85.6 | 101 | 14.4 | 0.99 | [0.76,1.30] |  |  |
| **Age at enrolment** | |  |  |  |  |  |  |  |  |
| <25 | 133 | 108 | 81.2 | 25 | 18.8 | 1.64 | [0.98,2.74] | 1.74* | [1.02,2.96] |
| 25-<40 | 1189 | 1010 | 84.9 | 179 | 15.1 | 1.26 | [0.92,1.72] | 1.41* | [1.02,1.96] |
| 40-<55 | 486 | 426 | 87.7 | 60 | 12.3 | Ref |  | Ref |  |
| ≥ 55 | 37 | 34 | 91.9 | 3 | 8.1 | 0.63 | [0.19,2.10] | 0.65 | [0.19,2.25] |
| **Place of residence** | |  |  |  |  |  |  | NS |  |
| Semi-urban | 310 | 217 | 70 | 93 | 30 | Ref |  |  |  |
| Rural | 731 | 667 | 91.2 | 64 | 8.8 | 0.22*** | [0.16,0.32] |  |  |
| Urban | 737 | 629 | 85.3 | 108 | 14.7 | 0.40*** | [0.29,0.55] |  |  |
| No data | 67 | 65 | 97 | 2 | 3 | 0.07*** | [0.02,0.30] |  |  |
| **Year of ART enrolment** | | |  |  |  |  |  | NS |  |
| 2016 | 124 | 110 | 88.7 | 14 | 11.3 | Ref |  |  |  |
| 2017 | 673 | 576 | 85.6 | 97 | 14.4 | 1.32 | [0.73,2.40] |  |  |
| 2018 | 450 | 373 | 82.9 | 77 | 17.1 | 1.62 | [0.88,2.98] |  |  |
| 2019 | 598 | 519 | 86.8 | 79 | 13.2 | 1.2 | [0.65,2.19] |  |  |
| **WHO stage at ART enrolment** | |  |  |  |  |  |  | NS |  |
| 1 | 1719 | 1502 | 87.4 | 217 | 12.6 | Ref |  |  |  |
| 2 | 77 | 64 | 83.1 | 13 | 16.9 | 1.41 | [0.76,2.60] |  |  |
| 3 or 4 | 13 | 12 | 92.3 | 1 | 7.7 | 0.58 | [0.07,4.46] |  |  |
| No data | 36 | 0 | 0 | 36 | 100 | NA |  |  |  |
| **Key population** | |  |  |  |  |  |  |  |  |
| FSW | 1003 | 855 | 85.2 | 148 | 14.8 | Ref |  | Ref |  |
| MSM | 522 | 444 | 85.1 | 78 | 14.9 | 1.01 | [0.75,1.37] | 1.15 | [0.84,1.57] |
| PWID | 316 | 276 | 87.3 | 40 | 12.7 | 0.84 | [0.58,1.22] | 1.01 | [0.68,1.49] |
| TG | 4 | 3 | 75 | 1 | 25 | 1.93 | [0.20,18.64] | 1.43 | [0.14,14.46] |
| **CBART approach** | |  |  |  |  |  |  |  |  |
| OSS | 865 | 744 | 86 | 121 | 14 | Ref |  | Ref |  |
| DIC | 554 | 532 | 96 | 22 | 4 | 0.25*** | [0.16,0.41] | 0.25*** | [0.16,0.40] |
| Outreach | 426 | 302 | 70.9 | 124 | 29.1 | 2.52*** | [1.90,3.35] | 2.59*** | [1.94,3.46] |

* p < 0.05, ** p < 0.01, *** p < 0.001 , Ref- reference

NS: not significant, VL – viral load test *OSS – One Stop Shop clinic, DIC – community drop-in-centre, FSW- female sex worker, MSM – men who have sex with men, PWID – person who inject drugs, TG – transgender people, OR-odd ratio, aOR, adjusted odd ratio, CBART- community-based antiretroviral therapy*
